# Hole Localization in Bulk and 2D Lead-Halide Perovskites Studied by Time-Resolved Infrared Spectroscopy

**DOI:** 10.1021/jacs.4c02958

**Published:** 2024-07-10

**Authors:** Daniel Sandner, Kun Sun, Anna Stadlbauer, Markus W. Heindl, Qi Ying Tan, Matthias Nuber, Cesare Soci, Reinhard Kienberger, Peter Müller-Buschbaum, Felix Deschler, Hristo Iglev

**Affiliations:** † Chair for Laser and X-ray Physics, Physics Department, TUM School of Natural Sciences, Technical University of Munich, James-Franck-Straße 1, 85748 Garching, Germany; ‡ Chair for Functional Materials, Physics Department, TUM School of Natural Sciences, Technical University of Munich, James-Franck-Straße 1, 85748 Garching, Germany; § Institute of Physical Chemistry, University of Heidelberg, Im Neuenheimer Feld 229, 69120 Heidelberg, Germany; ∥ Centre for Disruptive Photonic Technologies, The Photonics Institute, Nanyang Technological University, 21 Nanyang Link, 637371 Singapore

## Abstract

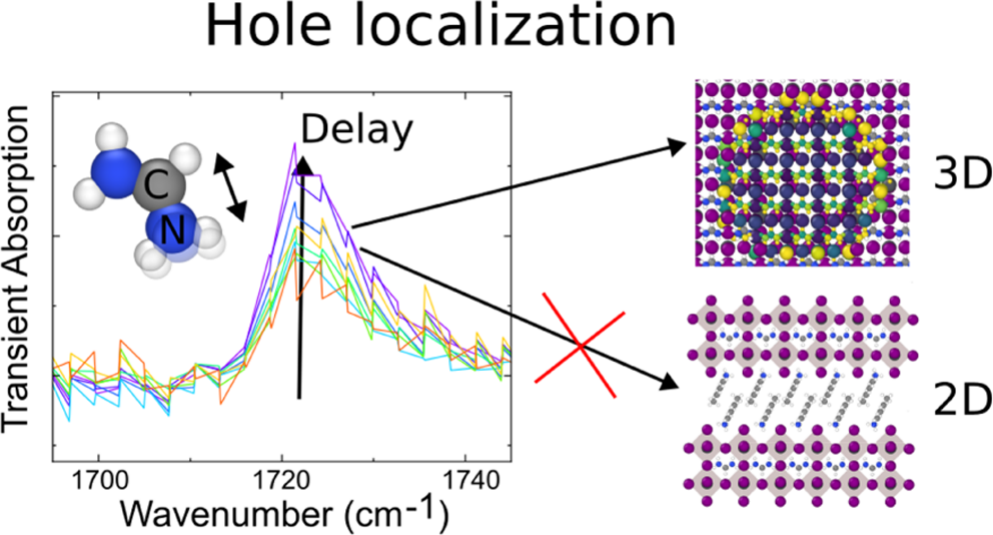

Scattering and localization dynamics of charge carriers in the soft lattice of lead-halide perovskites impact polaron formation and recombination, which are key mechanisms of material function in optoelectronic devices. In this study, we probe the photoinduced lattice and carrier dynamics in perovskite thin films (CsFAPbX_3_, X = I, Br) using time-resolved infrared spectroscopy. We examine the CN stretching mode of formamidinium (FA) cations located within the lead-halide octahedra of the perovskite structure. Our investigation reveals the formation of an infrared mode due to spatial symmetry breaking within a hundred picoseconds in 3D perovskites. Experiments at cryogenic temperatures show much-reduced carrier localization, in agreement with a localization mechanism that is driven by the dynamic disorder. We extend our analysis to 2D perovskites, where the precise nature of charge carriers is uncertain. Remarkably, the signatures of charge localization we found in bulk perovskites are not observed for 2D Ruddlesden–Popper perovskites ((HexA)_2_FAPb_2_I_7_). This observation implies that the previously reported stabilization of free charge carriers in these materials follows different mechanisms than polaron formation in bulk perovskites. Through the exploration of heterostructures with electron/hole excess, we provide evidence that holes drive the formation of the emerging infrared mode.

## 1 Introduction

Lead-halide perovskites (LHP) with the general structure APbX_3_ (X = Cl, Br, I) have attracted great interest as they combine the high luminescence yield^1^ and charge-carrier lifetime^2^ of monocrystalline semiconductors with the tunability of solution-processed organic semiconductors. The bandgap, for example, can be controlled by exchanging the A-cation or the halide to achieve current-matching conditions in tandem solar cells with silicon.^3^ Moreover, excitonic properties can be tuned by confinement, as introducing large organic spacer molecules causes self-assembled quasi-2D materials.^4^

The exceptional optoelectronic performance of LHPs is often attributed to charge-carrier-lattice interactions. The formation of large polarons is associated with stabilizing charge carriers as well as allowing long lifetimes and diffusion lengths in a defect-rich environment.^5,6^ Adversely, strain by large polarons may also be the driving force behind halide segregation, an irreversible process that causes degeneration in bandgap-tailored mixed-halide perovskite solar cells.^7^ While free charge carriers or polarons are generally considered the majority species in bulk (3D) perovskites, the nature of photoexcitations in 2D perovskites is yet to be explored. From the large exciton binding energy, exceeding the thermal energy (∼*k*_B_*T*) by far, excitons are expected to be the majority species.^8^ However, the analysis of radiative carrier recombination,^9,10^ ultrafast conductivity measurements,^11^ and tandem transient absorption/photoluminescence^12,13^ indicate that excitons dissociate fast into single charge carriers.

Polarons, being quantized charge-lattice interactions, are best observed via structural probes, e.g., X-ray diffraction or vibrational spectroscopy. Moreover, the formation occurs on the ultrafast femto-to-picosecond time scale, requiring appropriate methods. Time-resolved X-ray diffraction showed the expansive strain of large polarons^14^ and rotations of the lead-halide octahedra^15^ in the prototype system MAPbBr_3_ (methylammonium lead bromide), yet these experiments are challenging due to the destructive nature of the probe beam as well as typically larger pump fluences, compared to state-of-the art optical pump probe measurements. In addition, there are no systematic studies on the influence of temperature, composition, or confinement due to the limited availability of pulsed X-ray sources. Time-resolved infrared spectroscopy has been applied previously to study changes in structure as well as charge distribution. Transient absorption (TA) shows shifts of infrared modes as the bond length or charge distribution undergoes changes, as well as newly appearing peaks, for example, so-called infrared activated vibrations (IRAVs) by symmetry breaking and structural rearrangement in the presence of photoexcited charge carriers.^16,17^

Among the wide class of LHP, we choose formamidinium (FA) as A-cation due to its relevance for photovoltaic energy conversion and the high oscillator strength of a CN stretching vibration, making the FA cation a local reporter that has been selected before in studying charge-lattice coupling.^18−21^ In this work, we confirm an infrared mode, slightly blue-shifted from the CN mode, resulting from spatial symmetry breaking and study it using time-resolved infrared spectroscopy with subpicosecond time resolution and subwavenumber spectral resolution. The observed TA spectra can be decomposed into a peak–valley-shaped feature and the mode that increases within the first 100 ps after excitation. By studying heterostructures of the perovskite with electron/hole transport layers, we find evidence that hole polarons are observed. At low temperatures, we find strongly reduced polaron formation, which points toward the picture of dynamic disorder. Most notably, the experimental signature of polarons in bulk perovskites is not observed in quasi-2D perovskites with strong confinement. This contradicts localization as a mechanism for the stabilization of free carriers in 2D Ruddlesden–Popper (RP) perovskites.

## 2 Results and Discussion

**Figure 1:**
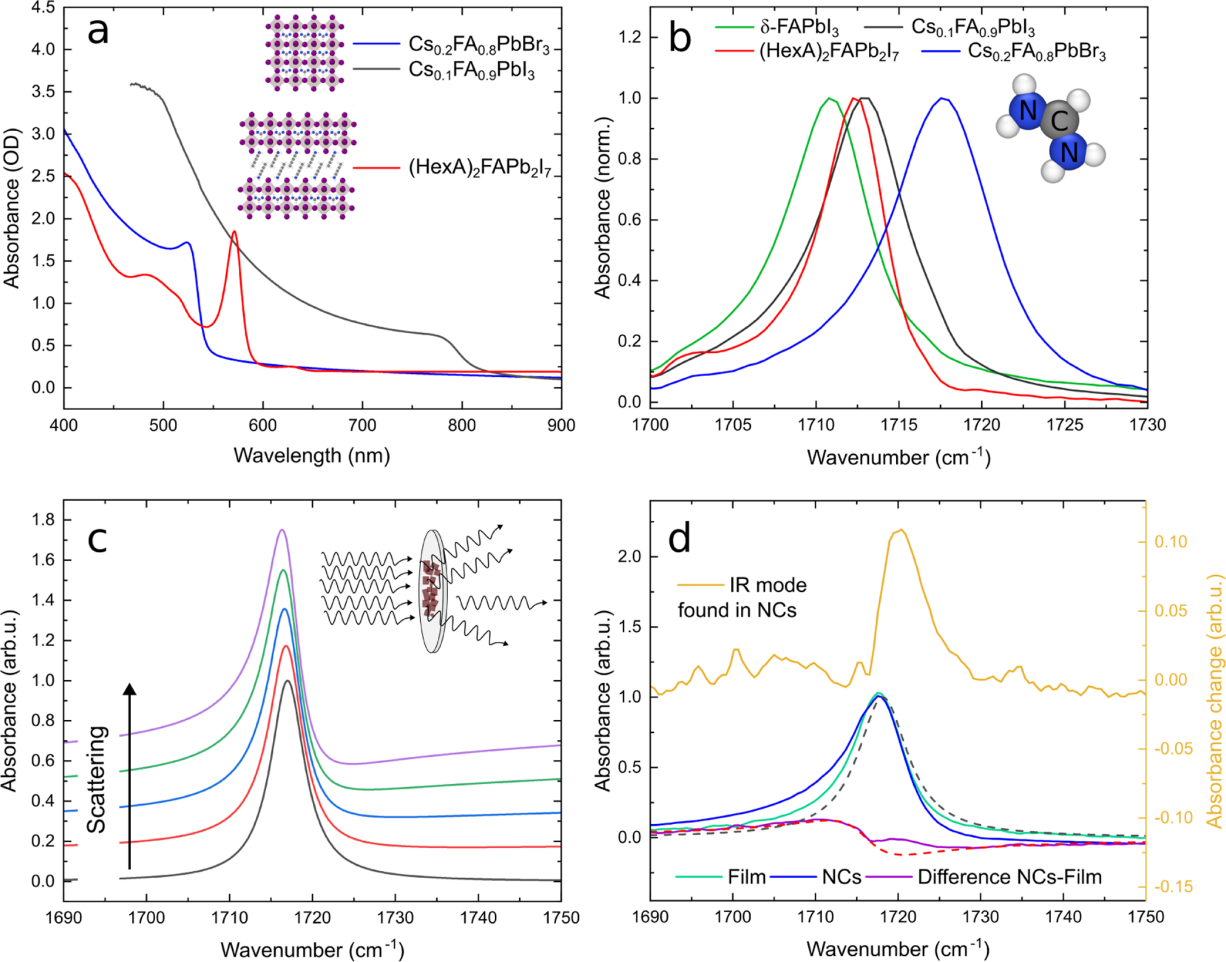
(a) UV–vis absorbance spectra of bulk (black and blue lines) and 2D (*n* = 2, red line) perovskite thin films. The inset shows the separation of perovskite layers by large organic spacers. (b) Infrared absorption spectra of thin films showing the CN stretching mode of the FA cation (schematically shown on the right side). (c) Model calculations showing the impact of scattering on the shape of IR absorption modes due to the Christiansen effect. (d) Comparison between IR spectra of Cs_0.2_FA_0.8_PbBr_3_ thin films (turquoise line), nanocrystals (NCs, blue), and a Lorentzian line shape (dashed black). The difference between the film and NCs can be separated into a peak–valley shape (dashed red curve) and an additional Lorentzian-like mode (see yellow line and right *y*-axis).

Absorption spectra of spin-coated films were measured in the UV and visible spectral regions to determine the quality of the polycrystalline films and suitable excitation wavelengths for pump–probe experiments (Figure 1a). More details on the sample fabrication and characterization can be found in Section S1. Small fractions of cesium were added to FAPbI_3_/Br_3_ to increase their stability. The spectral shape and position of the bandgap is in good agreement with the literature.^22^

From the bulk samples (blue and black curves), the strong influence of the halide on the bandgap becomes apparent. Smaller halides like Cl or Br cause a shrinking of the lead-halide octahedra and blue-shifted the absorption onset that is associated with the bandgap. In red, a 2D perovskite (*n* = 2) is shown. This means that two layers of lead-halide octahedra are each separated by the large organic spacer hexylammonium (HexA). The general formula changes from APbX_3_ for 3D perovskites to C_2_A_*n*–1_Pb*_n_*X_3*n*+1_ for 2D perovskites with a spacer group C. Compared to their bulk counterparts, the absorption onset is strongly blue-shifted, and an excitonic peak is observed below the bandgap energy.^23^

In Figure 1b, Fourier transform IR (FTIR) absorption spectra are shown around 1700 cm^–1^, where the CN stretching mode is located. The halide anions have a strong influence on the peak position as the FA cation (displayed in the inset of Figure 1b) is enclosed in the lead-halide cage. Moreover, structural changes, like the photoinactive delta phase of FAPbI_3_, manifest themselves as small shifts in the peak position.^24^ (HexA)_2_FAPb_2_I_7_ and Cs_0.1_FA_0.9_PbI_3_ show almost the same peak position, which implies that the lead-halide cage in 2D perovskites has a similar size compared to its 3D counterpart. A closer look at the infrared spectra shows a systematic deviation from a Lorentzian line shape. For all samples, the low-frequency tail is more pronounced than the high-frequency tail, especially at low temperatures where a dip appears at the high-frequency side (see Section S2). This effect is referred to as dispersion artifact^25^ or generally as the Christiansen effect,^26^ which describes the dependency of scattering on the refractive indices at the interface. The phenomenon arises in scattering samples, such as polycrystalline films or NCs, because the scattering cross section depends on the refractive index difference between a sample and its environment, which changes strongly close to a resonance.^27^ The magnitude of the effect strongly depends on the microstructure and, therefore, on the sample synthesis and the spectrometer geometry. Figure 1c shows modeled absorbance data for samples with different scattering strengths. The inset demonstrates that scattering appears as absorption when measuring the transmitted intensity. An extended comparison of the model and our measurements can be found in Section S2.

To study the influence of spatial symmetry breaking and charge gradients, we measured IR spectra of Cs_0.2_FA_0.8_PbI_3_ thin films and NCs with a mean size of 11 nm. Figure 1d shows the infrared spectrum of a thin film, NCs, and a Lorentzian peak. Note that IR peaks are often described by Gaussian, Lorentzian, or mixed spectral lineshapes, depending on the dominant relaxation and dephasing mechanisms.^28^ Because of the soft nature of the perovskite lattice,^29^ we describe the IR peaks in this work with a Lorentzian line shape, which has stronger wings compared to a Gaussian peak. Due to the Christiansen effect, both the polycrystalline film and NCs show additional absorption at the low-frequency tail and less absorption at the high-frequency tail, with the effect being more pronounced for NCs. The difference between the infrared spectra (NCs–film) deviates from a pure peak–valley line shape (see red dashed line), and the deviating peak is shown separately in yellow. We attribute this infrared peak, blue-shifted by ∼3 cm^–1^, to the breaking of centro-symmetry at the NC surface. Because of the disturbed periodicity and dangling bonds, the local structure is altered at the surface and the resonance frequency of the CN stretching is shifted. The relative amplitude of the additional peak is 0.1, while the fraction of unit cells in the surface layer of the NCs is 0.3. By considering that the additional IR mode is only observed when the specific bond is aligned with the surface vector and the polarization of IR light, we find that only 1/3 of the entire surface area contributes to IR absorption, in good agreement with the observed relative amplitude. In addition, the blue-shifted IR mode has not been observed for larger NCs with a size of 120 nm, which feature a surface-to-volume ratio of only 0.03 (see Section S6). We emphasize that the intramolecular vibrations, as C=N bonds, are sensitive to the local chemical environment and react via a blue shift to the softening of the surrounding structure.^30^ Note that due to the softer structure of the perovskite lattice, this symmetry breaking is not limited to the very last crystal layer but most likely also includes some neighboring layers. The small blue shift between Cs_0.1_FA_0.9_PbI_3_ and (HexA)_2_FAPb_2_I_7_ in Figure 1b can also be explained by the cesium incorporation, which reportedly causes structural distortions and a spatial symmetry breaking of the lattice.^31^

**Figure 2:**
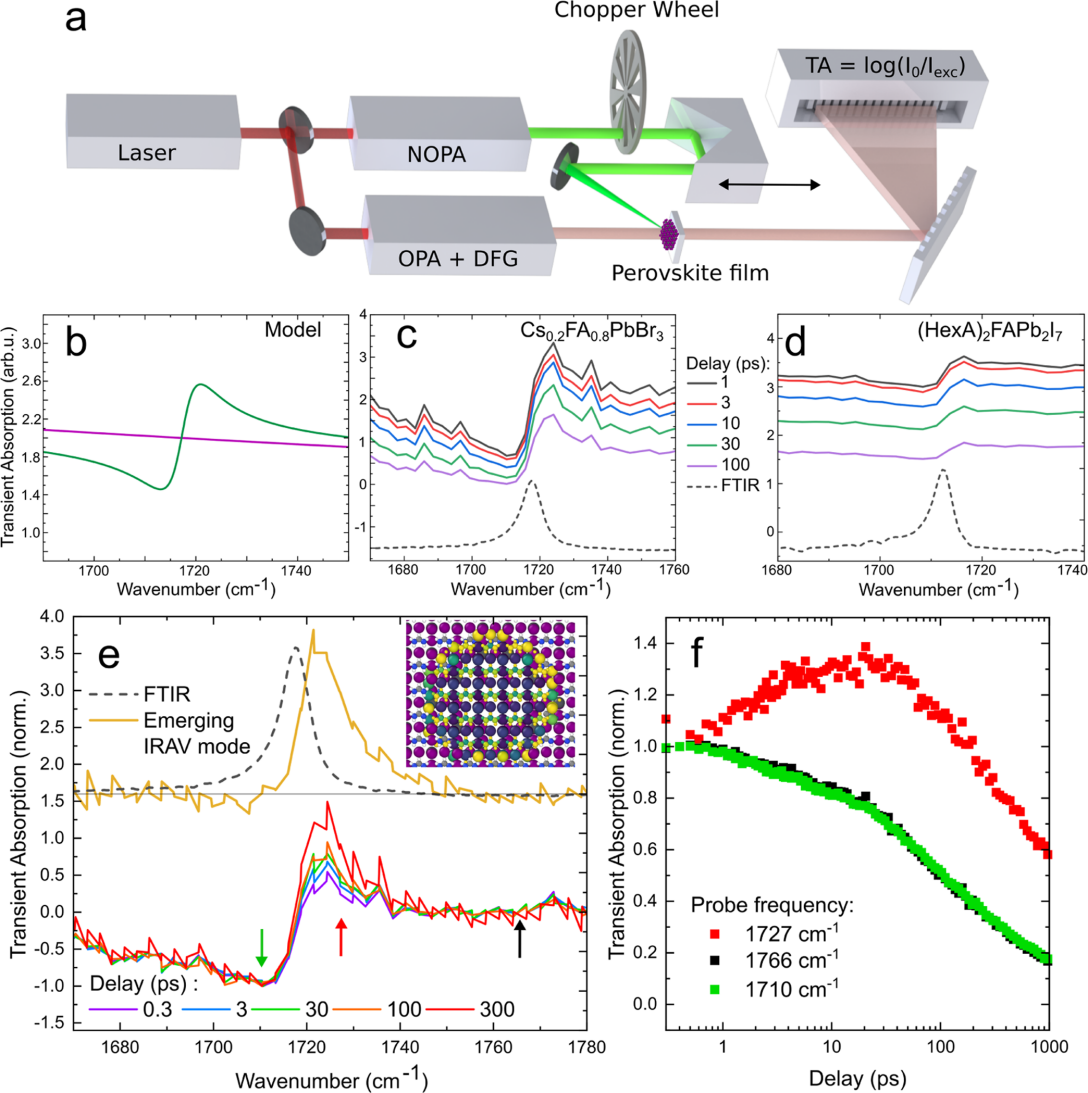
(a) Sketch of the TA setup used for time-resolved IR spectroscopy. The output of an ultrafast laser system is split into two beams where nonlinear optics is used to change the frequency to match the bandgap (green beam, pump) and the CN stretching mode (pale red bream, probe). The pump beam, which can be delayed, is overlapped at the sample with the probe beam, which is dispersed on a detector row. (b) TA around the IR mode with (green line) and without (purple) the Christiansen effect. (c, d) TA spectra at different delay times (see color code between c and d). Dashed lines show the FTIR spectrum. (e) TA spectra of Cs_0.2_FA_0.8_PbBr_3_ at various delays normalized to compensate recombination. The spectral shape changes with the delay and subtraction indicate that the yellow peak emerges over time. Green, red, and black arrows indicate the spectral positions of the transients shown in panel (f). Inset illustrates the breaking of centro-symmetry at the polaron surface. (f) Pump–probe dynamics of Cs_0.2_FA_0.8_PbBr_3_ at different spectral positions normalized at 0.4 ps.

After having discovered an emerging mode emerging by spatial symmetry breaking, we study the spectral region in a pump–probe experiment. Figure 2a depicts the TA setup. A single source of ultrashort laser pulses (amplified Ti:sapphire laser system, 800 nm, 1 kHz, 120 fs) is used and split in two beam paths to ensure subfemtosecond (fs) synchronization. A noncollinear optical parametric amplifier (NOPA) is used to generate pump radiation tunable in the range of 500–750 nm, while the IR probe pulses (1820–1600 cm^–1^) are generated by an optical parametric amplifier (OPA) and subsequent difference-frequency generation (DFG). Every second pump pulse is blocked by a mechanical chopper wheel; thus, comparison of adjacent infrared intensities yields the differential transmission/transient absorption caused by the optical excitation of the sample. The relative timing of pump and probe pulses is swept by a mechanical delay stage. IR spectra are acquired by dispersing the probe beam on a 64-pixel MCT detector row with a grating. A more detailed description of the pump–probe setup can be found in Section S3. Samples were always excited close to their energy gap (excess energy below 0.1 eV) to reduce the effect of hot carriers and an increase in lattice temperature. The pump fluence was in the range of 15–150 μJ/cm^–2^.

It has been previously reported that photoexcited charge carriers cause a positive transient absorption within 100 fs by intraband transitions, sometimes called polaron absorption.^32,33^ This induced, broad absorption has been shown to yield Fano-like interference with the narrow IR modes.^34^ Moreover, the broad absorption goes along with a change in the refractive index that diminishes scattering. Figure 2b shows the broad polaronic absorption in the absence of scattering (purple line) and the valley–peak shape expected for Fano-interference and scattering samples in which the dispersion artifact^27^ is modulated by the photoexcitation (green line). Indeed, as shown in Figure 2c,d for Cs_0.2_FA_0.8_PbBr_3_ and (HexA)_2_FAPb_2_I_7_, we observe a broad positive transient absorption with modulation at the position of the CN stretching mode (see dashed lines for the FTIR spectra). The valley–peak shape can also be interpreted as a blue shift of the IR mode due to solvatochromism.^35^ By the photoexcitation, the refractive index and dielectric function changes almost instantly (<100 fs),^36^ which alters the reaction field between the FA molecule and the surrounding lattice. The amplitude of the TA spectra decays by carrier recombination.

To study TA around the CN stretching vibration in detail, the spectra were normalized to a spectral position far away from the resonance followed by a subtraction of the broad background. The result is displayed in Figure 2e for a thin film of Cs_0.2_FA_0.8_PbBr_3_. One observes a valley–peak line shape at 0.3 ps that changes its shape between 1720 and 1740 cm^–1^ for increasing delay times. We extracted the emerging peak by subtracting an early TA spectrum from a late one and show the difference spectrum vertically shifted by the yellow line in Figure 2e. The horizontal black line indicates the corresponding zero line. As can be seen from the FTIR spectrum (black dashed line), the additional emerging peak in the late TA spectrum is blue-shifted by 5 cm^–1^. Figure 2f shows the pump–probe transients recorded at a fluence of 150 μJ/cm^2^ for several spectral positions (see arrows of the corresponding color in Figure 2e). The red and green curves show the dynamics of the peak (1727 cm^–1^) and valley (1710 cm^–1^) after subtraction of the background. The transients confirm that the positive TA signal at the high-frequency tail of the IR mode increases for tens of picoseconds while the polaronic absorption signal (and thus the carrier population) observed at 1766 cm^–1^ decays monotonously by Auger recombination, as shown in Section S8. We reported similar transients before for Cs_0.2_FA_0.8_PbBr_3_ and showed that the effect is independent of the pump fluence and, therefore, not linked to carrier–carrier interactions.^21^ The slow increase of the signal on the tens of picoseconds time scale indicates that this is not an electronic but a structural effect. Note that throughout the paper, we will use the term “free charge carrier” for both electrons and holes as the products of exciton dissociation and the term “localized charge carrier” when key properties of electrons or holes have been altered by the structural rearrangement following the interaction between the charge carrier and the lattice. Time-resolved XRD has shown that expansive strain fields emerge in MAPbBr_3_ after photoexcitation on a time scale of up to 100 ps.^14^ Similarly, ultrafast THz measurements indicate carrier localization–meaning an influence of a charge carrier on its own properties by deforming the polar lattice around itself–in FA_0.85_Cs_0.15_Pb(I_0.97_Br_0.03_)_3_ on this time scale as their effective mass increases up to 200 ps.^37^ Nishida et al. studied the broad transient absorption spectrum of a triple cation perovskite between 1100 and 1800 cm^–1^ and analyzed the data using a large polaron model and Drude theory, showing an evolution up to 100 ps in the polaron size and Drude scattering time, respectively.^38^ Due to the similar time scales and our observation in NCs (see yellow line in Figure 1d), we assign the blue-shifted, emerging mode to the spatial symmetry breaking around a localized charge carrier. The presence of a strong IR mode overlapping with the emerging mode implies that the activation of a previously IR-forbidden vibrational mode is not necessary to account for the observation. Instead, the transient spectrum can be explained by an amplitude modulation and a blue shift of the existing CN mode because of the (photoexcited) lattice distortions. Our observation of a vibrational response accompanying the electronic excitation goes beyond the approximations of Drude theory, but it is possible that the behavior of the dressed charge carriers can be described as Drude-like. Note that the far-field pump probe experiments reported herein average over a spot size of tens of micrometers, far exceeding the length scale of expected heterogeneities. Previous studies have found that the blue shift of the emerging mode^20^ as well as the ultrafast dynamics, including recombination and lattice response, can show strong local variations.^38^ All our time-resolved measurements should be treated as weighted average over many structural and compositional heterogeneities with varying dynamics and amplitudes.

Generally, the data in Figure 2e,f can be either interpreted as the decay of initial bleaching or the rise of absorption at 1727 cm^–1^. To distinguish these cases and support the assignment to localized charge carriers, we aim to tune the charge-carrier–lattice interaction by the lattice temperature. In conventional semiconductors, large polarons are described in the Fröhlich model, which relies on the coupling of charges to LO phonons in polar materials.^39^ While the Fröhlich model for polarons assumes small distortions from ideal lattice sites and therefore harmonic interactions, the picture of dynamic disorder can be applied to a soft, anharmonic lattice with large distortions, as it is expected for LHPs.^29^ Here, the lattice dynamics at finite temperatures are so strong that the electronic structure is affected by overlap fluctuations. Most notably, the model predicts reduced localization and larger mobilities at low temperatures.^29^ In contrast, strain fields arising from Coulomb interaction between the carrier and a charged ion in the lattice should persist even at low temperatures.

**Figure 3:**
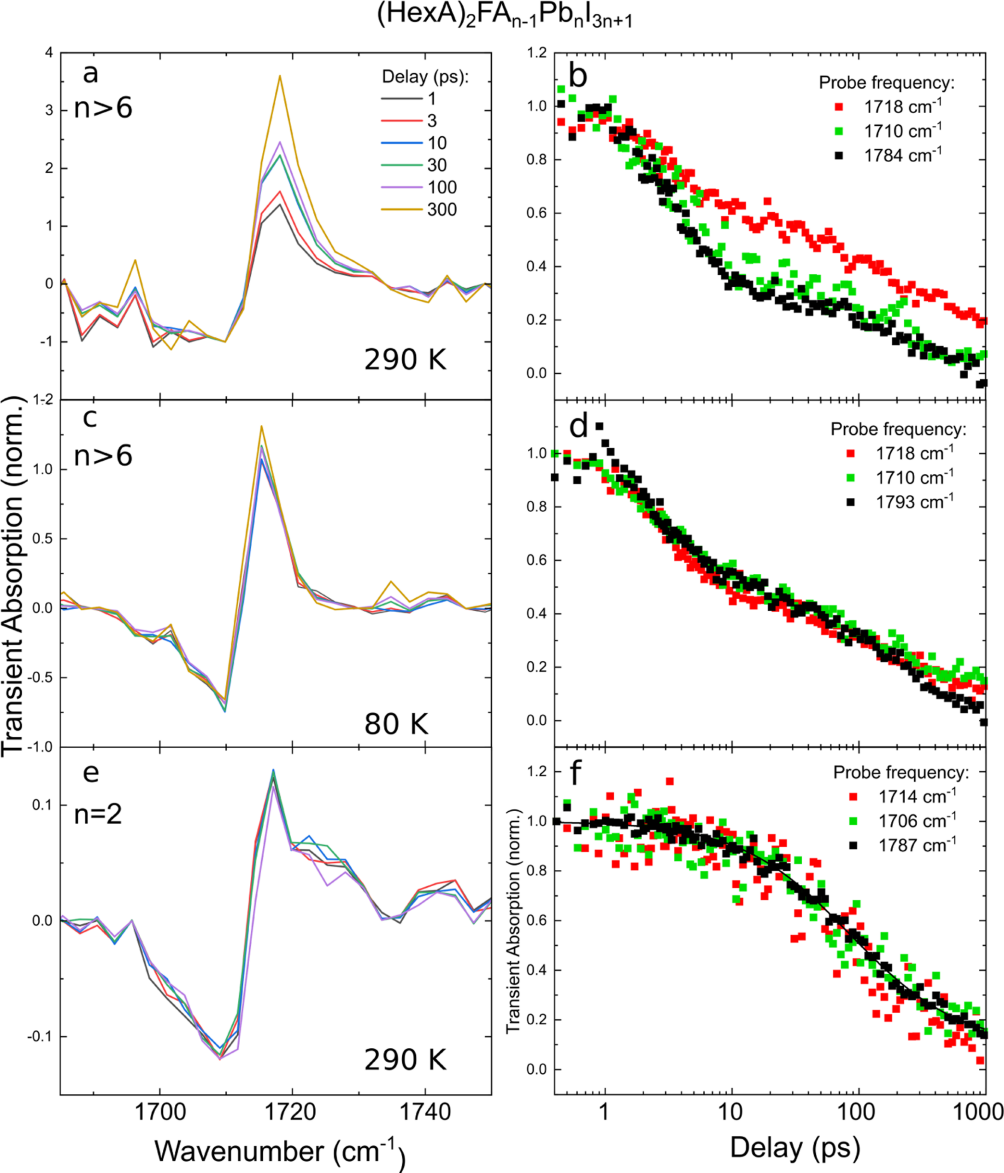
Left column (a, c, e): TA spectra normalized to the transient absorption signal far from the resonance at various delay times (see the color code in panel (a)). Right column (b, d, f): Pump–probe dynamics at the given spectral positions (red: emerging peak, green: valley, black: background, normalized at 0.4 ps).

Figure 3a–d shows transient absorption measured at bulk-like (*n* > 6) phases of a (HexA)_2_FA_*n*–1_Pb*_n_*I_3*n*+1_ sample at different temperatures. The pump fluence of 150 μJ/cm^2^ created an initial carrier density on the order of 10^19^ cm^–3^, making Auger recombination the dominant process. As the bandgap reduces with increasing numbers of perovskite layers between the organic spacer molecules, one can selectively excite the bulk-like thick perovskite slabs. UV–vis spectra of these samples can be found in Section S4.

At room temperature (Figure 3a,b), we observe the same behavior as in Figure 2e,f, namely, the growth of a peak at the high-frequency tail of the IR mode. Likewise, the transients at the high-frequency side (red curve in Figure 3b) show distinct dynamics. Compared to the previously shown transients, the red curve does not increase in absolute value since the decay rate by recombination dominates. The emerging IR mode also shows a shorter, distinct dynamic with a rise time of 4 ps, compared to bulk perovskites (τ = 18 ps). The extraction of time constants is described in Section S10. The faster localization response in weakly confined perovskite slabs is in good agreement to a previously reported comparison of Cs_0.2_FA_0.8_PbBr_3_ NCs and bulk films.^21^

The specific dynamic of the peak around 1720 cm^–1^ disappears when the sample is cooled to 80 K. As can be seen in Figure 3c,d, the line shape around the IR mode does not change significantly, and the transients at different spectral positions are identical. The observed disappearance of the blue-shifted IR mode indicates that localization is strongly reduced at low temperatures. This is in good agreement with previously reported temperature-dependent time-resolved infrared spectroscopy of the broad polaronic absorption,^32^ transport measurements,^40^ and supports the physical picture of dynamic disorder.^29^ Moreover, the valley–peak line shape in Figure 3c is almost symmetric, similar to the early spectra at 290 K (see Figure 3a), which indicates that additional IR absorption shown in Figure 2e at 1720 cm^–1^ emerges over time instead of a bleaching that decays over time.

After studying the impact of temperature on localization, we are interested in the effect of strong confinement. By the large exciton binding energy in 2D perovskites, bound electron–hole pairs are expected to be the majority species. Despite this, experimental evidence suggests that photoexcitations in 2D perovskites show properties of free charge carriers, e.g., bimolecular recombination^9^ and electric transport.^11^ This has led some researchers to attribute polaronic properties to excitons (polaron excitons)^41^ and others to propose that polaron formation gives free carriers the same energetic advantage as exciton formation, allowing the dissociation of an exciton into a polaron pair.^12^

Transient spectroscopy on the *n* = 2 phase in a 2D RP perovskite sample ((HexA)_2_FAPb_2_I_7_) should clarify the question about the presumed localization of free charge carriers in strongly confined perovskites. The data, recorded at 290 K, presented in Figure 3e,f are very similar to measurements of bulk perovskites at low temperatures (Figure 3c) and show no sign of localization at the IR mode. The peak–valley shape in the TA spectra does not change its form over time and can be accounted for by the Christiansen effect, as shown in Figure 2b. Also, the dynamics at different spectral positions are identical (see Figure 3f). This behavior of the strongly confined, layered perovskite (HexA)_2_FAPb_2_I_7_ is also observed at 80 K (see Section S9). The background dynamics can be well described by bimolecular recombination (see the black line in Figure 3f and the discussion in Section S8), which matches the initial carrier density of ∼1.3 × 10^18^ cm^–3^. We want to discuss our findings in the context of other vibrational probes applied to (quasi) 2D perovskites. Zhang et al. used ultrafast electron diffraction and observed the ordering of the lead-halide octahedra by rotation into a more symmetric phase, but only for Dion–Jacobson (DJ) 2D perovskites.^42^ In contrast to Ruddlesden–Popper (RP) perovskites, DJ perovskites feature an initial distortion that is lifted by the photoexcitation.^42^ Therefore, similar to our study, no systematic structural change was observed in RP samples except for heating of the lattice by the high pump energy (3.1 eV) and fluence (few mJ/cm^2^).^42^ In our experiments, these heating effects are absent as the samples are excited resonantly at the exciton transition (2.2 eV) and with a much smaller pump fluence (20 μJ/cm^2^). Cuthriell et al. used ultrafast transient X-ray diffraction and predominantly found an expansion in the *c*-axis, meaning the spacing between perovskite layers increases on the hundreds of picoseconds time scale.^43^ As demonstrated in Figure 1b, the CN stretching mode is sensitive to the lead-halide bond length but not to the presence of organic spacers, as the CN mode looks very similar for Cs_0.1_FA_0.9_PbI_3_ and the 2D counterpart (HexA)_2_FAPb_2_I_7_. Therefore, we assume that our time-resolved measurements are not sensitive to changes along the *c*-axis. In good agreement with our experiments, both studies have not found evidence for a light-induced, symmetry-changing phase transition.^43^ This observation certainly does not exclude the presence of free carriers in quasi-2D perovskites but challenges the proposed mechanism of (large) polaron formation. The formation of small polarons cannot be excluded by our data, as this process can occur as fast as 400 fs;^44^ however, one could expect a more distinct vibrational response due to the large distortions. In addition, the small Stokes shift observed for (HexA)_2_FAPb_2_I_7_ contradicts strong self-trapping.^45^

Our measurements on 2D perovskites demonstrate that the emerging mode is specifically sensitive to the localization of free charge carriers in 3D perovskites but does not probe electrons or holes specifically. Previous studies could not determine whether the observed strain field of large polarons is generated by electrons or holes.^14^ Due to the opposite charge, the lattice deformations should differ from electron polarons to hole polarons. By studying CsPbBr_3_ NCs with transient X-ray absorption spectroscopy, Santomauro et al. concluded that electrons are delocalized and holes are localized as small polarons on bromide sites.^46^ Meanwhile, Österbacka et al. predicted the formation of small electron polarons via molecular dynamics simulations of the same material.^47^

**Figure 4:**
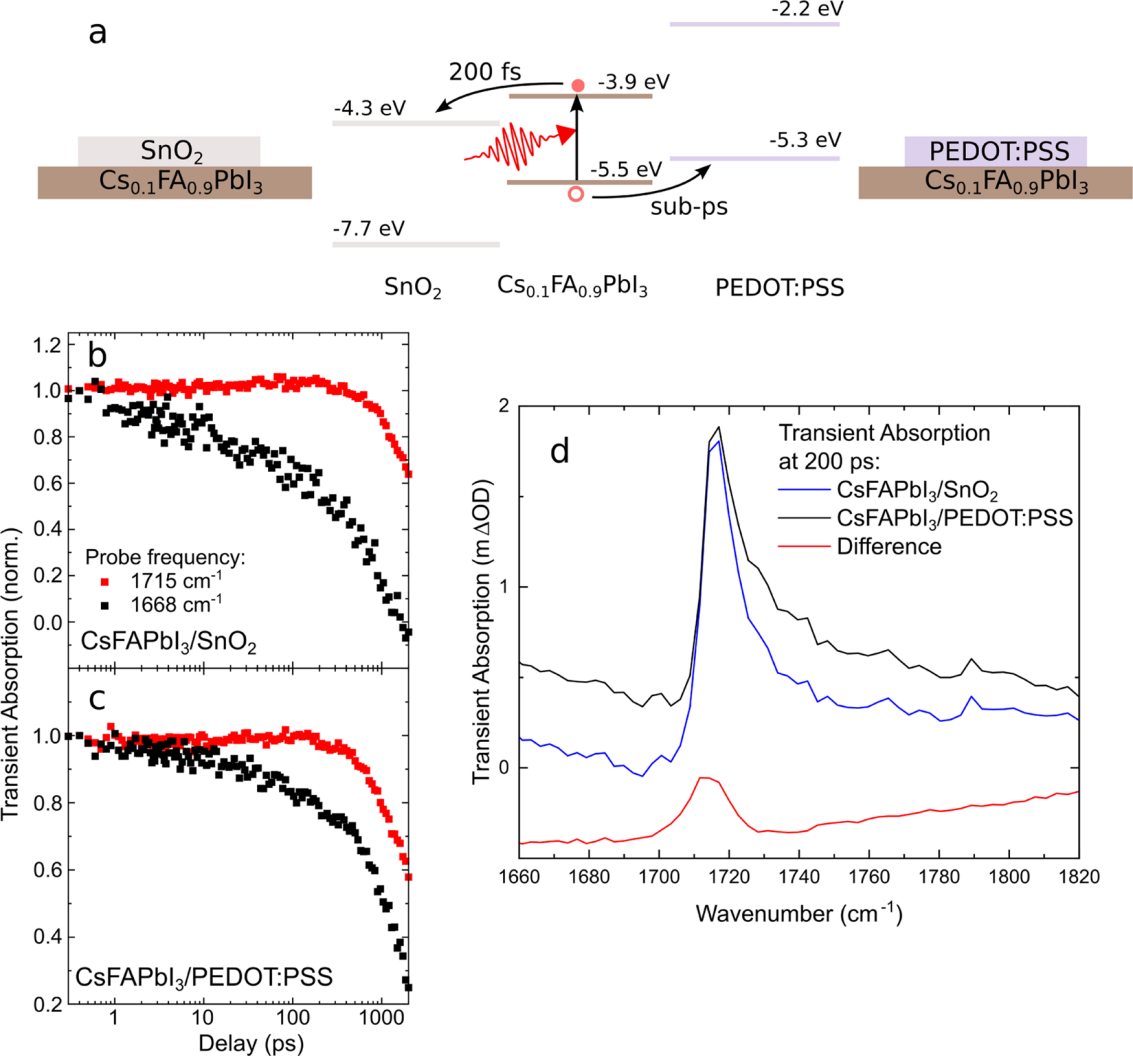
(a) Band alignment of Cs_0.1_FA_0.9_PbI_3_, SnO_2_, and PEDOT:PSS. Optically excited electrons and holes have been reported to transfer in the respective layers on a subpicoseconds time scale. (b, c) Transients at the emerging peak position (red) and background (black) for both heterostructures, normalized at 0.4 ps. (d) Transient absorption spectra of the different heterostructures at a pump–probe delay of 200 ps. The red line shows the spectrum of Cs_0.1_FA_0.9_PbI_3_/SnO_2_ subtracted by the spectrum of Cs_0.1_FA_0.9_PbI_3_/PEDOT:PSS.

The main obstacle in studying the ultrafast dynamics of electrons and holes individually is that the optical pump pulse excites electron–hole pairs. Doping is a very effective tool to study electrons or holes as the majority species, but it only allows steady-state measurements and cannot be used to monitor picosecond dynamics. To overcome this limitation, we performed pump–probe spectroscopy on type II aligned heterostructures, schematically shown in Figure 4a. This means that both the conduction and valence bands of a semiconductor are situated relatively higher than the conduction and valence bands of another semiconductor. While the pump pulse generates equal electron and hole densities in the perovskite, one of the species is driven into an acceptor material by the band offset. The heterostructures were made of Cs_0.1_FA_0.9_PbI_3_ with electron (SnO_2_) or hole (PEDOT:PSS) transporting layers (for more details, see Section S5). Importantly, the SnO_2_ and PEDOT:PSS layers are transparent at the pump frequency and do not show infrared modes around 1720 cm^–1^. Thus, only the CN stretching mode of the perovskite is observed.^48^Figure 4a shows the band alignment of the three materials according to the literature.^49,50^ The ultrafast, subpicoseconds charge transfer toward the electron transporting layer^50^ (ETL) and hole-transporting layer^51,52^ (HTL) enables us to study the effect of excess holes or electrons in the perovskite layer on a picosecond time scale as the other species has been partially removed.

Transient dynamics at the emerging peak position and the background are shown in Figure 4b,c for heterostructures between the perovskite and ETL(SnO_2_) and HTL (PEDOT:PSS), respectively. The pump fluence was 15 μJ/cm^2^, and the decay can be fully described by radiative band-to-band recombination (see Section S8). The proposed signature of polaron formation, the distinct dynamics of the high-frequency tail of the CN stretching mode (1715 cm^–1^), is observed for both samples. This implies that either electrons and holes show the same spectral feature or incomplete transfer of the respective species. As the perovskite films had a thickness of roughly 300–400 nm, only a fraction of the excited charge carriers can migrate into the ETL or HTL layer. Previously reported ultrafast transport measurements indicate that the carrier cannot diffuse across the entire film thickness of our samples within a few hundred picoseconds.^53^ Moreover, the heterostructures in our experiments are not contacted, so we expect the buildup of an electric field by the charge transfer that counteracts the band offset and hinders further depletion of one selected species. In summary, the heterostructures may show an excess electron or hole concentration but not a complete depletion of one species.

Figure 4d shows the as-measured TA spectra at a delay of 200 ps without background subtraction. Two samples were prepared for each heterostructure, and the TA spectra were averaged. To study the effect of small changes in the carrier concentration, we subtract the measurements made on both types of heterostructures from each other. Since the sample with the ETL shows an excess of holes, ETL-HTL data should yield the transient spectrum of holes (and the inverse transient spectrum of electrons). By subtracting the signal measured in Cs_0.1_FA_0.9_PbI_3_/PEDOT:PSS (HTL) from that in Cs_0.1_FA_0.9_PbI_3_/SnO_2_ (ETL) we obtain the red curve, which can be decomposed in a broad negative background and Lorentzian peak. The broad negative TA signal in the difference spectrum shown in Figure 3d indicates that the broad polaronic or free carrier absorption is altered in the heterostructure. More importantly, the presence of a peak at ∼1715 cm^–1^ in the difference spectrum indicates that the Cs_0.1_FA_0.9_PbI_3_/SnO_2_ sample had a higher concentration of the relevant species for the emerging peak observed in Figure 2e than the samples of Cs_0.1_FA_0.9_PbI_3_/PEDOT:PSS. Since the hole-transporting PEDOT layer extracts holes from the perovskite layer, we conclude that the emerging infrared mode is sensitive to the localization of holes. Due to the incomplete charge transfer, we cannot prove that the vibrational signal is exclusively evoked by holes. However, the significant difference in peak height (25% of peak height) at moderate differences in charge carrier concentration support a predominant influence of holes on the CN stretching peak. While our observation of localized holes is in good agreement with theoretical studies of polaron formation in CsPbBr_3_^54^ and smaller holes than electron mobility in CsFAPbI_3_,^55^^55^ it is still possible that electrons form polarons, too, without having an impact on the CN stretching mode of the FA cation.

## 3 Conclusions

In this work, we establish a spectroscopic tool to study predominantly hole localization in FA-based lead-halide perovskites and applied it to study the effect of the lattice temperature and strong confinement. We show that scattering samples can cause a derivative line shape at the CN stretching mode and such an observation is not necessarily a sign of structural change. Instead, we identify an emerging peak, which is slightly blue-shifted, as a sign of localization and find strong evidence through the study of heterostructures that hole localization is responsible for this emerging peak. When samples are cooled to cryogenic temperatures, the emerging peak disappears, indicating delocalized charge carriers at low temperatures. We apply our measurements to strongly confined 2D perovskites and find no evidence for hole localization. This suggests that free charge carriers, if present in 2D perovskites, are stabilized by different mechanisms compared to their 3D counterparts. We are convinced that our method can also be applied to study the localization of holes in lead-free perovskites.

## Supporting Information Available

Model calculations on Mie scattering; sample preparation; experimental methods; additional UV–vis and FTIR absorbance spectra; transient absorption spectra of heterostructures. (PDF)
