# Supplementary material for: Hole Localization in Bulk and 2D Lead-Halide Perovskites Studied by Time-Resolved Infrared Spectroscopy
Source: J Am Chem Soc. 2024 Jul 10;146(29):19852–62. doi: 10.1021/jacs.4c02958 (PMC11273617; doi:10.1021/jacs.4c02958)
Supplement: Supplementary file 1 — ja4c02958_si_001.pdf [file ja4c02958_si_001.pdf]

Hole localization in bulk and 2D lead halide perovskites studied by time-resolved infrared spectroscopy

*Daniel Sandner[1], Kun Sun[2], Anna Stadlbauer[3], Markus W. Heindl[3], Qi Ying Tan[4], Matthias Nuber[1], Cesare Soci[4], Reinhard Kienberger[1], Peter Müller-Buschbaum[2], Felix Deschler[3], and Hristo Iglev[1,]**

[1] Chair for Laser and X-ray Physics, Physics Department, TUM School of Natural Sciences, Technical University of Munich, James-Franck-Str. 1, 85748 Garching, Germany

[2] Chair for Functional Materials, Physics Department, TUM School of Natural Sciences, Technical University of Munich, James-Franck-Str. 1, 85748 Garching, Germany

[3] Institute of Physical Chemistry, University of Heidelberg, Im Neuenheimer Feld 229, 69120 Heidelberg, Germany

[4] Centre for Disruptive Photonic Technologies, The Photonics Institute, 21 Nanyang Link, Nanyang Technological University, Singapore 637371

- Corresponding authors: E-Mail: hristo.iglev@tum.de

Contents

Section S1: Synthesis and characterization of perovskite films/NCs....................3
Section S2: Christiansen Effect in IR spectroscopy....................4
Section S3: Implementation of time-resolved infrared spectroscopy....................6
Section S4: Characterization of mixed-phase quasi-2D perovskites....................7
Section S5: Characterization of perovskite heterostructures....................8
Section S6: Surface-to-volume ratio in perovskite NCs....................9
Section S7: Estimation of excited carrier density: 10
Section S8: Analysis of background dynamics....................11
Section S9: Transient IR spectra of 2D perovskites at low Temperatures....................16
Section S10: Quantifying the growth of the IR mode....................17
References....................18

Section S1: Synthesis and characterization of perovskite films/NCs

Chemicals used in the synthesis of $Cs_{0.2}FA_{0.8}PbBr_3$ quantum dot film/ bulk film:

Cesium bromide (CsBr, 99.9%), formamidinium bromide (FABr, ≥ 98%), lead (II) bromide ($PbBr_2$, 98%), oleylamine (OAm, technical grade 70%), dimethyl sulfoxide (DMSO, 99.9%), N,N-Dimethylformamide (DMF, 99.8%), and chlorobenzene (99.8%) were purchased from Sigma-Aldrich. Oleic acid (OA, technical grade 90%), and octadecene (ODE, technical grade 90%) were purchased from Alfa Aesar.

Synthesis of $Cs_{0.2}FA_{0.8}PbBr_3$ bulk film:

0.2 g of FABr, 0.08 g of CsBr, and 0.7 g of $PbBr_2$ were dissolved into 1.67 mL of DMSO until complete dissolution. Chlorobenzene was first spin-casted onto a calcium fluoride (CaF_2) substrate followed by the precursor solution at 4000 rpm. The resulting film was baked at 100°C for an hour. All synthesis were carried out in a nitrogen environment.

Synthesis of $Cs_{0.2}FA_{0.8}PbBr_3$ nanocrystal film:

0.2 g of FABr, 0.008 g of CsBr, and 0.7 g of $PbBr_2$ were dissolved in 5 mL of DMF. After complete dissolution, 50 µL of OAm and 100 µL of OA were subsequently added into the solution. 200 µL of the precursor solution was added into 5 mL of toluene and left to stir for 24 hours. The solution was drop-casted onto a CaF_2 substrate. All synthesis were carried out at room temperature.

TEM Images and Size distribution of $Cs_{0.2}FA_{0.8}PbBr_3$ NCs are already published in Figure 1 in ref (1).

Synthesis of n = 2 HexAFAPbI films:

The CaF_2 substrates used for the spin-coated thin film samples with $(HexA)_2FAPb_2I_7$ were first cleaned for 3 min in an ultrasonic bath in acetone and isopropanol at an reduced power of 70 W and afterwards put in an oxygen plasma for 5 min.

A 0.1 molar solution was achieved from precursor powders of Hexylammonium Iodide ($C_6H_{16}IN$), Formamidinium Iodide (CH_5IN_2) and Lead Iodide (PbI_2), which were dissolved in 2 ml Dimethylformamid (DMF). This solution was mixed at 300 rpm with a stirring bar while being heated on a 85°C hotplate for two hours. Afterwards, the solution was filtered through a PTFE-syringe filter with a pore diameter of 0.2 µm. On the cleaned CaF_2substrate, 120 µl of the solution were spin-coated with 5000 rpm for 25 sec. with a ramp of 1500 rpm. As fast as possible, the spin-coated films were put on a 140°C warm hotplate and kept there for three to five minutes. Finally, the samples, were taken from the hotplate and stored in the dark in sample boxes under nitrogen atmosphere.

Synthesis of HexFAPbI n = 2, 3 and beyond:

Perovskite samples with Hexylammonium-spacers were synthesized by dissolving 0.1383g of Lead(II)-Iodide (Sigma-Aldrich, 99.999%), 0.0344 g of Formamidinium Iodide (dyenamo, 99.99%) and 0.0458 g Hexylammonium Iodide (Sigma-Aldrich, n.s.) in 1 mL of Dimethyl Sulfoxide (Sigma-Aldrich, 99.9%). The resulting solution was filtered and 160 µL were spin-coated for 40 s at 6000 rpm on a CaF_2 substrate preheated to 140°C. The resulting film was subsequently annealed for 10 min. at 140°C.

Synthesis of $Cs_{0.1}FA_{0.9}PbI_3$ /SnO_2; PEDOT:PSS heterostructures:

Materials: Caesium iodide (CsI), formamidinium iodide (FAI), lead iodide (PbI_2), Dimethylformamide (DMF, 99.8%, anhydrous), dimethyl sulfoxide (DMSO, 99.9%, anhydrous), and chlorobenzene (99.9%, anhydrous) were purchased from Sigma-Aldrich. Tin (IV) oxide solution (SnO_2 in 15% H_2O colloid dispersion) and Poly(3,4-ethylene-dioxythiophene) Polystyrene Sulfonate (PEDOT:PSS, Al 4083) were purchased from Alfa Aesar and Ossila, respectively.

The substrates were treated by O_2 plasma for 10 min, and then the diluted tin (IV) oxide solution (SnO_2 : DI water = 1 : 4) or PEDOT: PSS solution (filtered) was spin coated on the substrates in air at 4000 rpm for 30 s and followed by annealing at 150 °C for 30 min. Afterwards the as-prepared samples were transferred into glovebox for the subsequent deposition. The perovskite layer (the precursor was prepared according to the stoichiometry of $Cs_{0.1}FA_{0.9}PbI_3$ in mixed solvents (DMF:DMSO = 4:1), where the concentration of $Pb^{2+}$is 1.0 M) was deposited onto the SnO_2 or PEDOT:PSS layer at 4000 rpm for 30 s and chlorobenzene (CB) was applied 10 s before ending. The samples were then annealed at 150 °C for 20 min.

Section S2: Christiansen Effect in IR spectroscopy

Scattering was modeled with the python implementation of the code of Bohren und Huffmann[2] that follows classical Mie theory. The refractive index and absorption around the resonance are described in the Lorentz oscillator model.

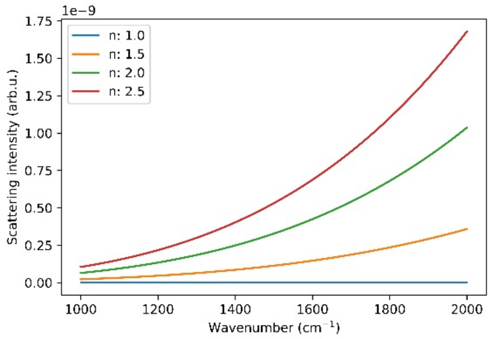

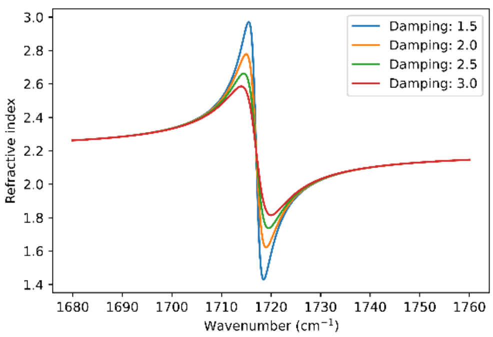

Figure S1 left: Scattering increases with frequency (wavenumber) and depends on the difference in refractive index between the material and the environment. At the interface to air (n = 1), the scattering cross section increases with the refractive index of the sample. Right: Change of refractive index around a vibrational mode by the Lorentz oscillator model.

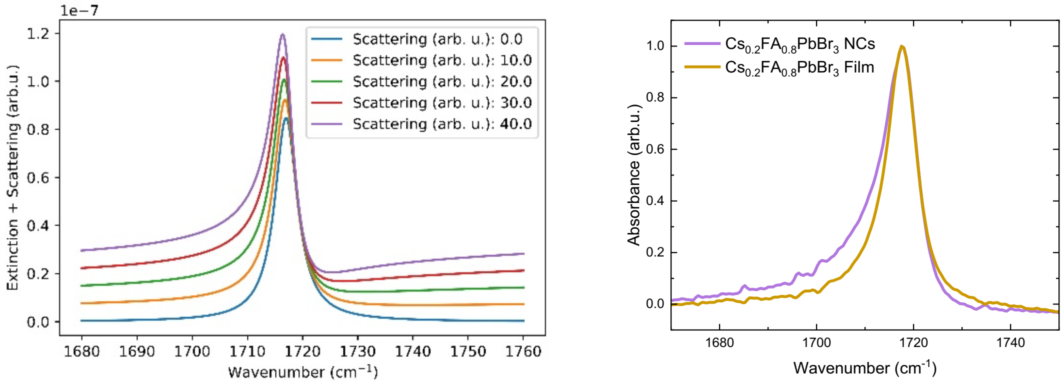

Figure S2 left: Increasing scattering adds a baseline absorption (when measured in transmission). Moreover, the absorption by scattering is not flat at the resonance but follows the refractive index in shape. In sum, the low frequency side is higher while the high frequency side shows lower absorption than the baseline. Right: Perovskite NCs show more scattering than a polycrystalline film due to the increased surface area. Therefore, the dispersion artefact is more pronounced. The absorption at the low frequency tail is larger than the absorption of the film while the opposite is the case for the high frequency tail.

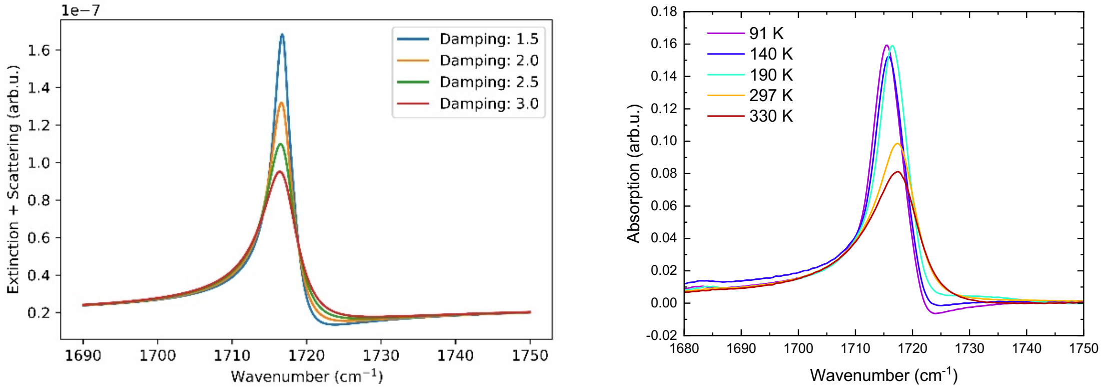

Figure S3 left: A change in the damping at the same oscillator strength changes the difference between minimal and maximal refractive index around the resonance. Therefore, the dispersion artefact is more pronounced for smaller damping. Right: This behavior can be seen in temperature dependent Absorption measurements as the linewidth (damping) is reduced as smaller temperatures.

Section S3: Implementation of time-resolved infrared spectroscopy

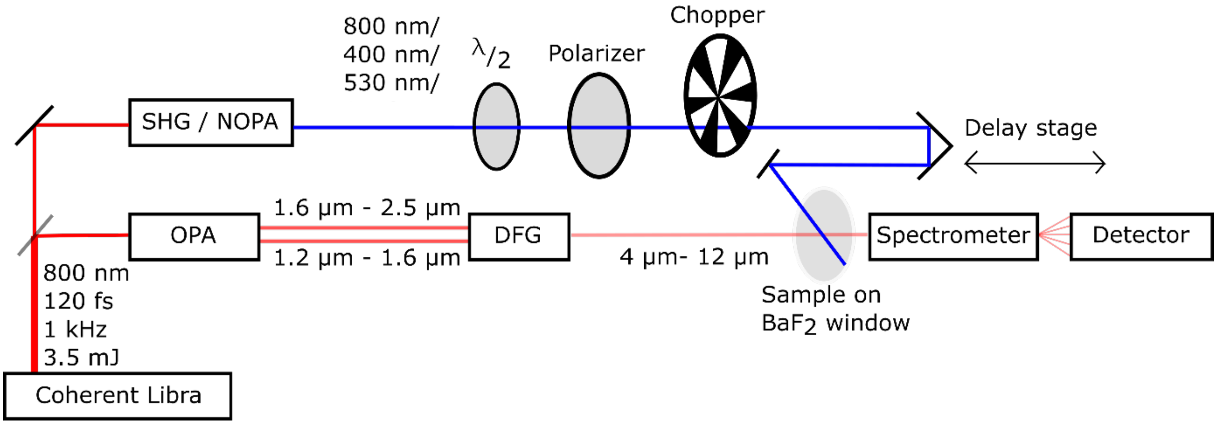

Figure S4: Sketch of the visible pump mIR-probe setup.

Figure S4 shows the used setup. The output of a Coherent Libra at 800 nm is split into a pump and a probe beam. In the pump beam, a homebuilt (design by E. Riedle) non-collinear optical parametric amplifier (NOPA) is used to generate light tunable between 500 and 750 nm. A lambda/2 waveplate and a polarizer are used to continuously adjust the pump power without introducing delay. A chopper at 500 Hz blocks every second pump pulse. A mechanical delay stage (folded twice) is used to vary change the relative pathlength and can introduce a relative time difference of up to 3 ns between pump and probe pulse.

The probe pulse in the mid IR is generated by difference frequency generation (DFG) between two beams generated as signal and idler in a homebuilt optical parametric amplifier (OPA). The probe beam is split into two beams called probe and reference to have a measure for the shot-to-shot noise (not shown in sketch). Pump and probe pulses are focused onto the sample with spot-sizes of roughly 200 and 50 µm, respectively. Probe and reference beam are spatially dispersed by a grating onto a 2x64 pixel MCT detector (infrared associates). Atmospheric water vapor is used for calibration. For each delay 4000 laser shots are acquired, 2000 with excitation and 2000 without. The delay range is multiple times swept and the scans are averaged. The magnitude of the transient signals stayed constant over many scans indicating non-destructive excitation and complete recovery of the sample within 1 ms.

For measurements at cryogenic temperatures the sample was mounted in a vacuum chamber and the sample holder cooled by liquid nitrogen.

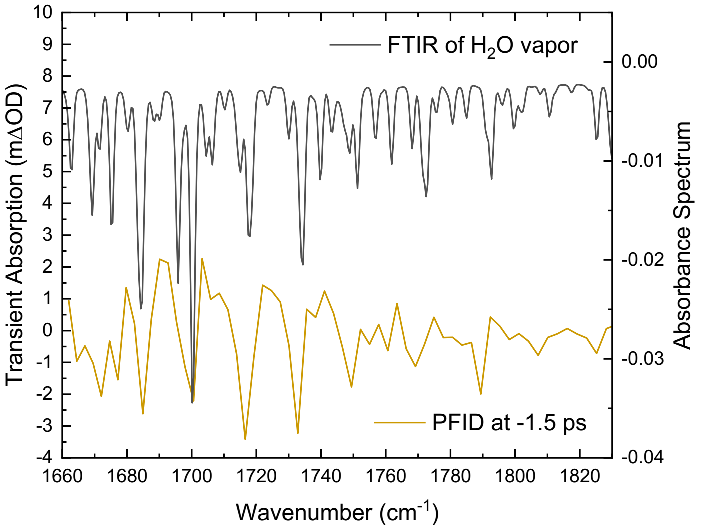

Figure S5: The FTIR spectrum of water vapor matches the observed perturbed free induction decay, confirming spectral accuracy within few cm^{-1}.

Section S4: Characterization of mixed-phase quasi-2D perovskites

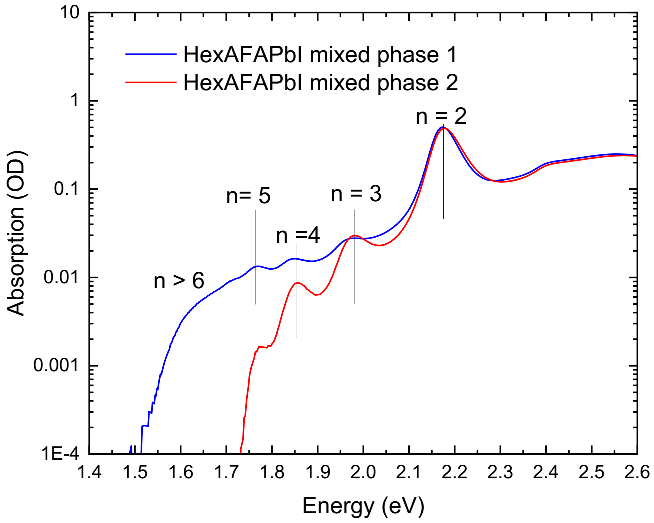

Figure S6: Uv-vis absorption of mixed-phase quasi-2D perovskites. One can clearly recognize the excitonic peaks of the different phases up to n = 4.

Figure S6 shows mixed-phase perovskites with bulk like contributions (n > 6, very close to the bandgap of bulk $FAPbI_3$). Scattering was extrapolated from the nIR region and subtracted. By using a pump energy of 1.65 eV we can selectively excite carriers in the bulk-like phases.

Section S5: Characterization of perovskite heterostructures

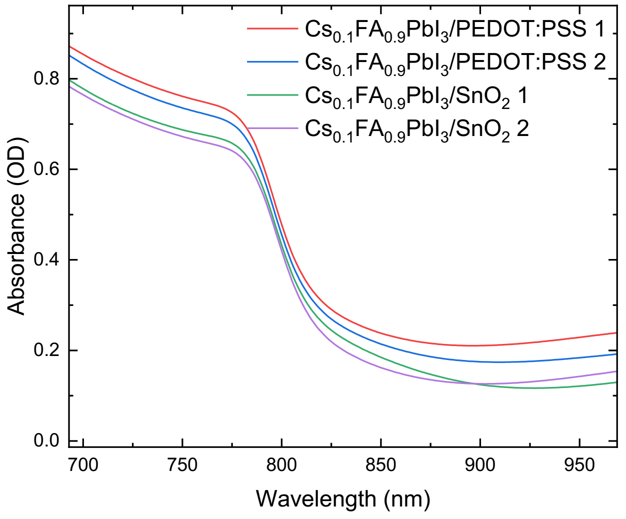

Figure S7: Uv-vis absorbance of all 4 heterostructures.

Figure S7 shows that the absorption increase across the bandgap (from 850 nm to 750 nm) is very similar for all 4 samples. This indicates that the perovskite film thickness is comparable and therefore the expected transient absorption signal under identical excitation. To avoid systematic effects, the samples were measured time-resolved in alternating order.

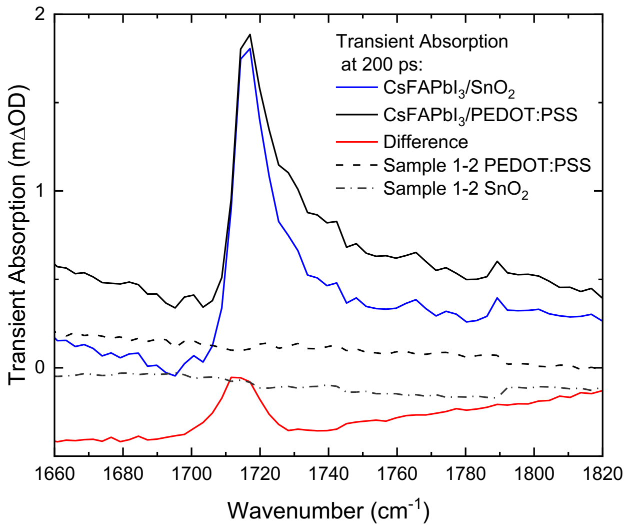

Figure S8: Transient spectra at 200 ps showing $Cs_{0.1}FA_{0.9}PbI_3/SnO_2$ (blue), $Cs_{0.1}FA_{0.9}PbI_3$/PEDOT:PSS (black) and the difference (red) (same data as in Fig 4d). In addition, the dashed lines show the difference between the two samples of the same kind. One can see that they are basically flat. Therefore, the difference between the different heterostructures cannot be explained by spread between individual samples.

Section S6: Surface-to-volume ratio in perovskite NCs

Assuming a cubical nanocrystal made of cubic unit cells, the fraction of unit cells exposed to the surface is $(n^3 - (n-2)^3)/n^3$. For a nanocrystal of 11 nm and a cubic unit cell parameter of 0.6 nm, we obtain ca. 19 unit cells along each edge. Therefore, the outer layer of unit cells corresponds to 28% of the entire NC volume. The amplitude of the additional IR mode in perovskite NCs is weaker (around 10% of the mode at 1717 cm^{-1}). The factor of 1/3 between the amplitude of the emerging IR mode and the total surface area can be understood as follows: Since IR spectroscopy is bond sensitive, the effect of the surface, e.g., an electric field, is only observed if the bond (e.g., the NCN backbone) is aligned with the vector normal to the surface. However, IR bonds only absorb light when the dipole moment is parallel to the light polarization. In the illustration, all four molecules are aligned along the surface vector however only those in the green marked area can absorb the incident light. In summary, only 1/3 of the total surface area can contribute to the IR signal (see schematic illustration).

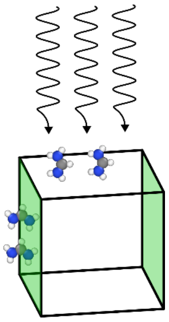

To further exclude the influence of additional reactants in the NC synthesis, we studied the FTIR spectra of 120 nm-sized NCs (roughly 200 unit cells in size). The surface layer only accounts for 3% of the NC volume.

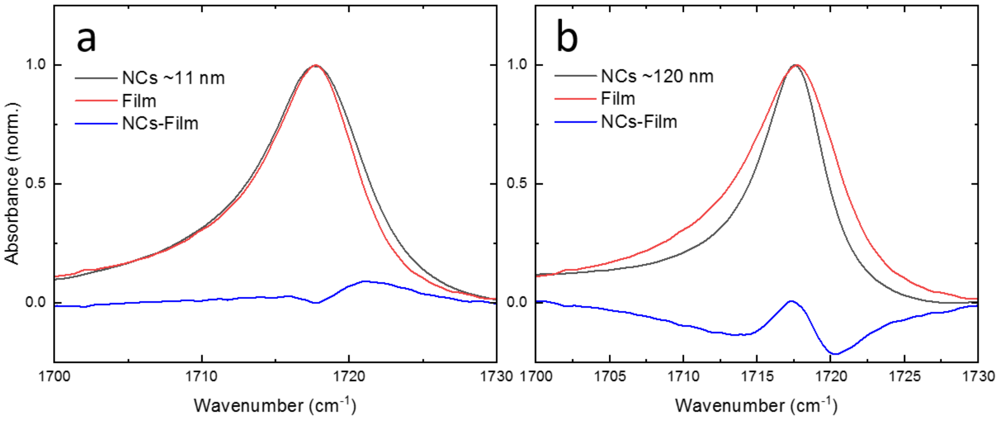

Figure S9: FTIR spectra (normalized to peak maximum) of $Cs_{0.2}FA_{0.8}PbBr_3$ NCs of size ~11 nm (a) and 120 nm (b). The thin film, made of the same composition, was spin-coated. The blue curves show the difference between the NCs and the thin film. For NCs of 11 nm size (S9 a), the difference shows another peak blue-shifted by ~ 4 cm^{-1}. This effect is not observed for larger NCs, as shown in Fig. S9b. Because of the smaller spectral width of the large NCs, the difference is negative at the flanks of the peak. Most notably, the high-frequency side is lower than the low-frequency side, indicating no other IR peak on the high-frequency side.

The smaller spectral width of the IR mode in 120 nm sized NCs indicates a more homogeneous growth of the NCs in solution compared to the crystallization process of the thin film occurring

during spin-coating. An AFM image of the thin film shows that it is polycrystalline with a broad distribution in grain size.

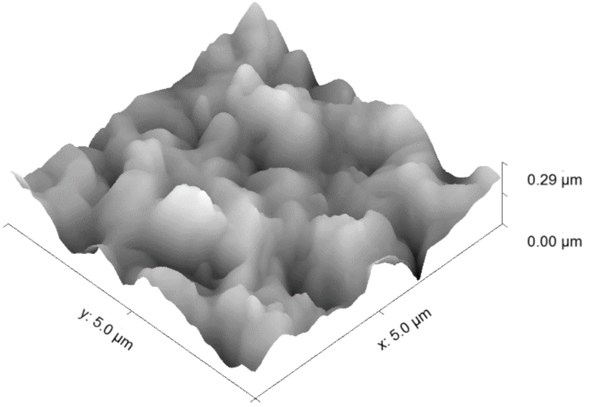

Figure S10: AFM image of the $Cs_{0.2}FA_{0.8}PbBr_3$ thin film.

Section S7: Estimation of excited carrier density:

We want to estimate the density of electron-hole pairs after excitation (*n*) in a thin film with thickness d, based on the pump fluence (F).

The photon flux F_{ph} that can be absorbed by the sample is given by the fraction of the incident light F that is not reflected at the interface (1-R), divided by the photon energy.

$$F_{ph} = \frac{F\,(1-R)}{E_{ph}}$$

The amount of light that is absorbed can be calculated using the Lambert-Beer's absorption law. The intensity of a beam of light I_0 is attenuated over a distance d in a material with an absorption coefficient of α as follows:

$$I(d) = I_0 e^{-\alpha d}$$

The fraction of photons absorbed by the layer of thickness d is given by $[I_0 - I(d)]/I_0$. For small values of αd we can write:

$$\eta_{abs} = \frac{I_0 - I(d)}{I_0} = (1 - e^{-\alpha d}) \approx \alpha d$$

The carrier density is given by the number of excited electron-hole pairs $N_{e\text{-}h}$ per volume V:

$$n = \frac{N_{e-h}}{V} = F_{ph}\alpha d$$

In the case of the data presented in Figs. 4b and 4c, the pump fluence was 15 $\mu J/cm^2$ at a photon energy of 1.65 eV. In the literature, the reflectivity of $FAPbI_3$ is reported to be 0.15 [3,4] . We therefore obtain an effective photon flux of $4.8\cdot10^{13}$ cm^{-2}. The absorption coefficient can be estimated from the linear absorbance A: A = α·d. From the absorbance data presented in Fig. S7 we extract an absorbance coefficient of $2.4\cdot10^{4}$ cm^{-1}, in agreement with literature[4]. Finally, we obtain a carrier density of $1.2\cdot10^{18}$ cm^{-3} for a pump fluence of 15 $\mu J/cm^2$.

Section S8: Analysis of background dynamics

For all samples, we observed a broad, positive transient absorption signal in the mid-IR that evolved within our instrument response function and decayed monotonously afterwards (see black dots in Figs. 2f, 3b, 3d, 3f, 4b and 4c). The analysis of these transients can reveal the dominant recombination mechanisms and indicates the carrier density in the sample. In the literature, carrier dynamics in perovskites have been often described by a multi-exponential fit. All transients recorded by us could be fitted with reasonable quality by three or more exponential terms. However, the lifetime of the transient signals shortened for larger pump fluences in our experiments. This contrasts with the model of exponential decay. For an exponential decay, the lifetime is expected to be independent of the carrier density n since the decay rate depends linearly on n ($\frac{dn}{dt} = -kn$ → n(t) = e^{-kt}).

For carrier densities as excited in pump-prob experiments, one must often consider the interactions between photoexcitations (electrons, holes, excitons etc.). Interactions involving 2 carriers are called bimolecular and can be radiative recombination or Exciton-Exciton annihilation (EEA). Recombination involving 3 charge carriers is typically called Auger recombination. Since the decay rates of these effects increase with the carrier density, the mean lifetime is shortened at larger pump fluences.

We fitted bimolecular decay with the established function: $TA(t) = A \cdot n(t) = \frac{An_0}{n_0 k_2 t+1}$ (A is a scalar describing the transient signal of a single photoexcitation, n_0 stands for the initial charge carrier density and k_2 the coefficient for bimolecular recombination[5]. One can see that this fulfills the differential equation: $\frac{dn}{dt} = -k_2 n^2$. Note that in the fit process, the carrier density and radiative recombination rate have a high interdependence. Therefore, the estimated carrier densities (see Section S7) have been used as starting parameters.

<u>$Cs_{0.1}FA_{0.9}PbI_3$:</u>

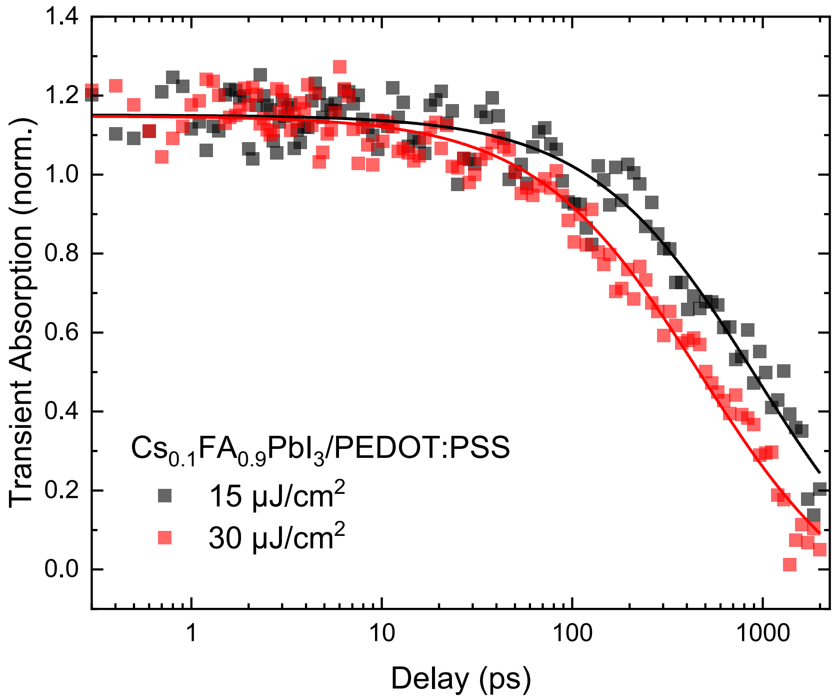

Figure S11: Transient absorption recorded at 1668 cm^{-1} (background) for $Cs_{0.1}FA_{0.9}PbI_3$/PEDOT:PSS heterojunctions (c.f. Fig 4c). One can see from the normalized transients (dots) that a larger pump fluence causes a shorter lifetime. The bimolecular fit (solid lines) shared the coefficient k_2 for both curves and extracted $k_2 = 8 \cdot 10^{-10}$ cm^3s^{-1} and carrier densities of $1.3 \cdot 10^{18}$ and $2.6 \cdot 10^{18}$ cm^{-3} for the pump fluences of 15 and 30 µJ/cm^2, respectively. The carrier density is in good agreement with the calculation presented in Section S7. Since the model predicts the carrier densities to be in the same ratio as the pump fluence, we are confident that bimolecular recombination is the dominant decay mechanism. Since free carriers are expected for the bulk perovskite, $Cs_{0.1}FA_{0.9}PbI_3$, the decay channel would be radiative band-to-band recombination of electrons and holes.

$(HexA)_2FAPb_2I_7$:

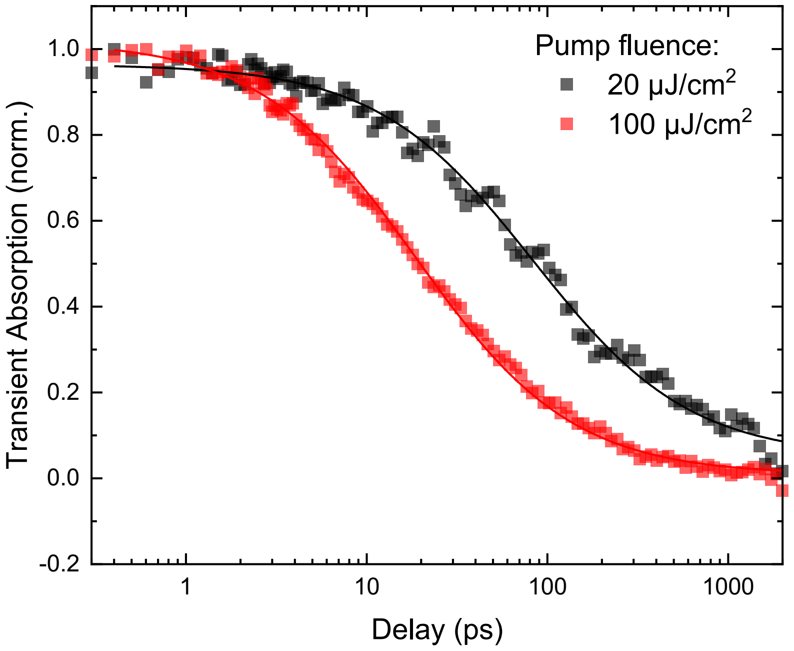

Figure S12: Transient absorption of $(HexA)_2FAPb_2I_7$ for a probe frequency of 1787 cm^{-1} (c.f. Fig 3f). The data (dots) has been fitted with a bimolecular function (solid lines). The parameters are $k_2 = 1.4 \cdot 10^{-9}$ s^{-1}, n = $8.6 \cdot 10^{18}$ cm^{-3} and $3.8 \cdot 10^{19}$ cm^{-3} for pump fluences of 20 and 100 $\mu J/cm^2$, respectively. The higher carrier density in 2D perovskites compared to 3D perovskites can be explained by the differences in the absorption coefficient. Like our analysis of dynamics in $Cs_{0.1}FA_{0.9}PbI_3$ (see Fig. S11), the fit reproduces approximately the ratio of the pump fluences.

$Cs_{0.1}FA_{0.9}PbBr_3$:

Transient absorption measurements presented in Figs. 2e, 2f and 3a-d were acquired under a larger pump fluence (~100 µJ/cm^2) to obtain a better S/N. Consequently, the model of bimolecular recombination fails to describe the full curve as higher order effects like Auger recombination need to be considered. The fit functions in the time domain would need to fulfill the differential equation:

$$\frac{dn}{dt} = -k_1 n - k_2 n^2 - k_3 n^3$$

The solutions of these differential equations can get rather complicated. It is more useful, to study the decay rate directly by differentiating the transient absorption curves. Before differentiating, curves were smoothed. The method is demonstrated for a TA measurement of $(HexA)_2FAPb_2I_7$, that has been analyzed already using a bimolecular fit (c.f. Fig S11).

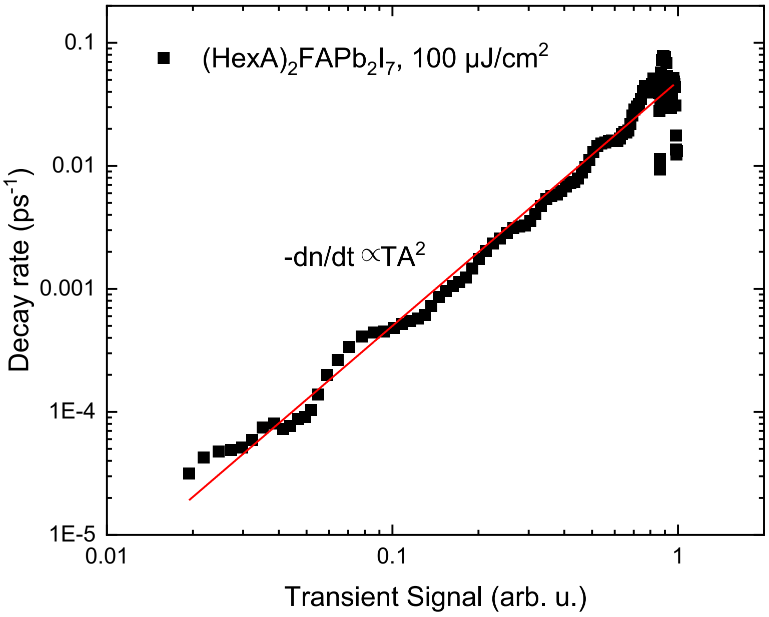

Figure S13 shows the decay rate $(-\frac{dTA}{dt})$ versus the transient signal, that serves as proxy for the carrier density, of $(HexA)_2FAPb_2I_7$. A linear fit with slope 1.98 in the logarithmic plot shows that the decay rate depends on the carrier density squared and supports the validity of the bimolecular model used in Fig. S11.

Mixed-phase samples of (HexA)FAPbI were also excited with a larger fluence (~ 100 µJ/cm^2) to yield a larger S/N since the bulk-like phase ($n > 6$) makes up only a small fraction of the sample. The dynamics also feature a shorter mean lifetime which indicates Auger or higher order effects.

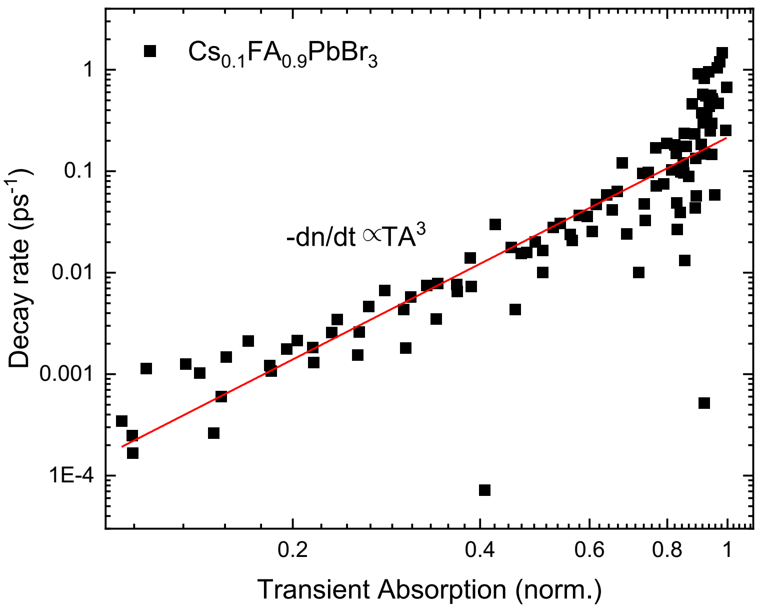

Figure S14 shows the decay rate versus the transient signal for $Cs_{0.1}FA_{0.9}PbBr_3$ excited at ~150 µJ/cm^2. After a fast initial drop, due to higher order effects, the curve can be well described in a log-log Plot by a line of slope 3.1 ± 0.2. This indicates Auger recombination as dominant recombination channel. The initial carrier density is expected to be on the order of 10^{19} cm^{-3}.

Section S9: Transient IR spectra of 2D perovskites at low Temperatures

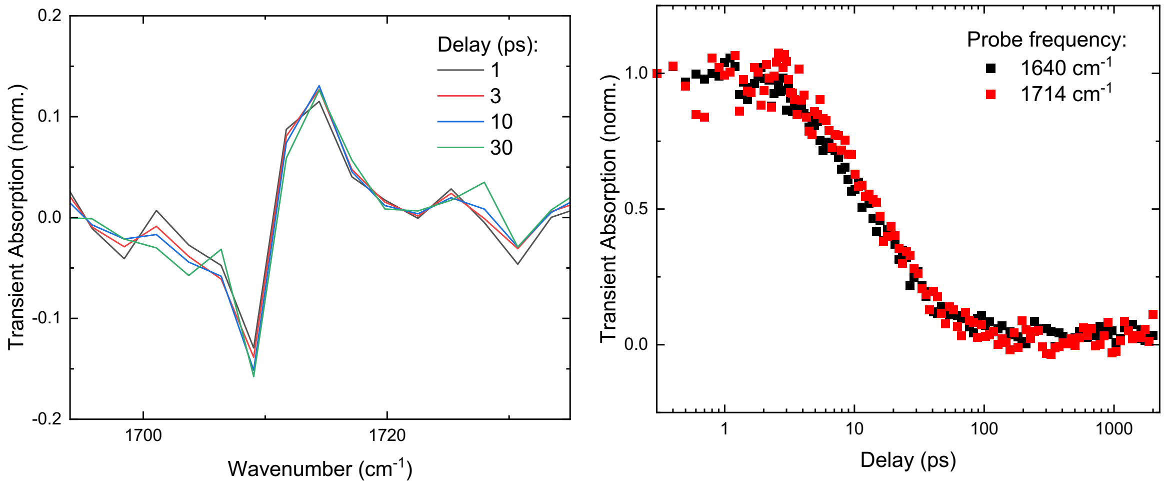

Figure S15 left side: Transient IR spectra, normalized to the TA signal at 1640 cm^{-1}, of $(HexA)_2FAPb_2I_7$ at 80 K (c.f. Fig. 3e in the main text). Right side: Transients at the peak position (1714 cm^{-1}) and a spectral position far away from the IR mode (1640 cm^{-1}).

One can see that similar to Figs. 3c-f, a valley-peak line shape is observed that doesn't change it's shape and decreases in amplitude by carrier recombination.

Section S10: Quantifying the growth of the IR mode

A shown in Figs. 2f, 3b, 4b, 4c; we observe an additional time-dependent component in bulk perovskites at room Temperature. To describe the risetime of this signal, that we attribute to polaron formation, we need to deconvolute the growth of the signal with the overall decay. In the following we assume that the broad IR background by free carrier absorption is linear proportional to the carrier population n(t) with a constant factor of C. The overall signal decays because of carrier recombination, and so does the transient lattice response, as reported before[6]. On timescales exceeding the polaron formation, both the background signal and the TA signal at the IR mode show the same decline since the polaron formation function converges to P_{max}. We normalized the curves at a late delay (multiplying $TA_{Background}$ by a coefficient B such that $A + P_{max} = B$) and subtracted the background from the IR mode. This yields the polaron growth function $P_{formation}(t)$ multiplied by the time-dependent carrier density n(t). Therefore, the difference between the TA signal at the IR mode and the background was divided by the background signal. The remaining term vanishes as $P_{formation}(t)$ saturates and contains no other time-dependent variables.

$$TA_{Background} = C \cdot n(t)$$

$$TA_{IR\ mode}(t) = [A + P_{formation}(t)] \cdot n(t)$$

$$\frac{TA_{IR\ mode}(t) - B \cdot TA_{Background}}{C \cdot n(t)} = \frac{A + P_{formation}(t) - B}{C}$$

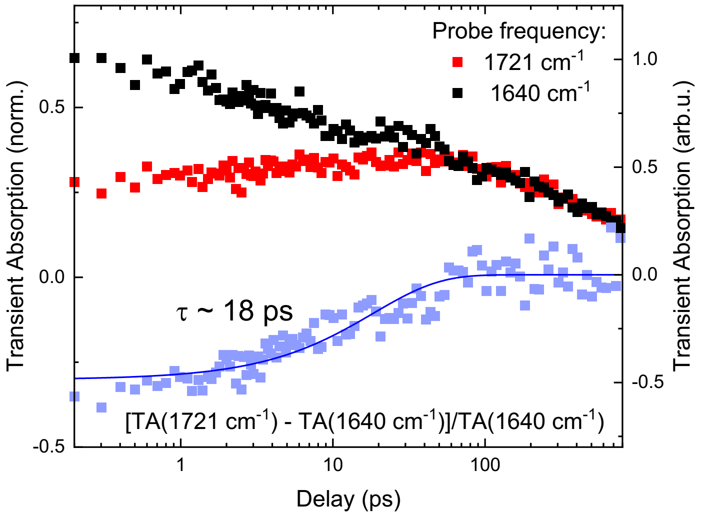

Figure S16: TA transients of a $Cs_{0.2}FA_{0.8}PbBr_3$ film normalized in the range of 500 ps (left y-axis). On the right y-axis, the difference between these curves, divided by the TA signal at 1640 cm^{-1} is shown (blue dots). The curve can be described by a single exponential process with a time constant of 18 ps.

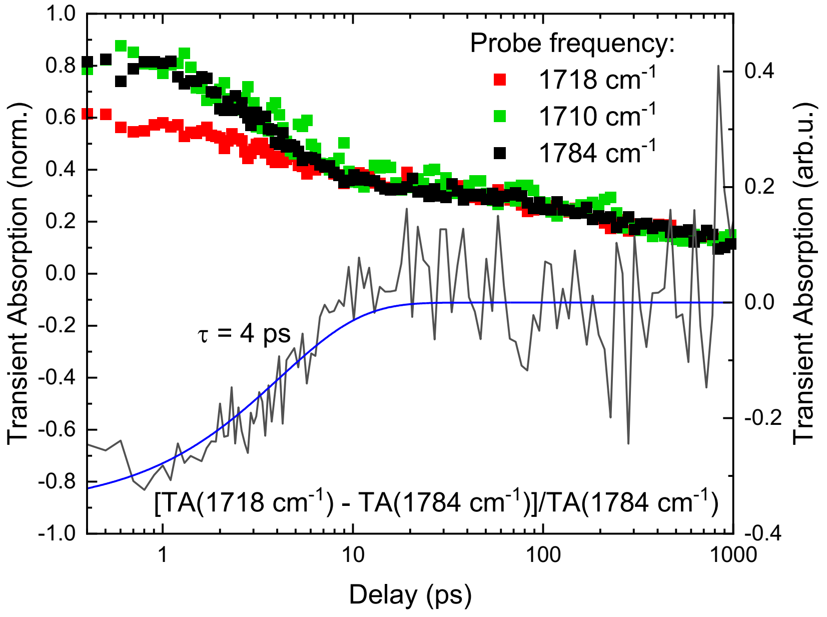

Figure S17: TA transients of (HexA)FAPbI (n>6) normalized at 100 ps shown on the left y-axis (same data as in Fig. 3b). The rising signal of the emerging IR mode (black line, right y-axis) can be described by a monoexponential term with decay constant of 4 ± 1 ps.

The faster growth of the IR signal observed in slabs of (HexA)FAPbI compared to bulk perovskites ($CsFAPbBr_3$, $CsFAPbI_3$) is in agreement to a previous report on faster polaron formation in CsFAPbBr NCs[1].

References

(1) Nuber, M.; Tan, Q. Y.; Sandner, D.; Yin, J.; Kienberger, R.; Soci, C.; Iglev, H. Accelerated polaron formation in perovskite quantum dots monitored via picosecond infrared spectroscopy. *J. Mater. Chem. C* **2023**, *11* (10), 3581–3587. DOI: 10.1039/D2TC04519B.

(2) Bohren, C. F.; Huffman, D. R. *Absorption and Scattering of Light by Small Particles*; Wiley, 1998. DOI: 10.1002/9783527618156.

(3) El-Ghtami, H.; Laref, A.; Laref, S. Electronic and optical behaviors of methylammonium and formamidinium lead trihalide perovskite materials. *J Mater Sci: Mater Electron* **2019**, *30* (1), 711–720. DOI: 10.1007/s10854-018-0340-2.

(4) Kojić, V.; Bohač, M.; Bafti, A.; Pavić, L.; Salamon, K.; Čižmar, T.; Gracin, D.; Juraić, K.; Leskovac, M.; Capan, I.; Gajović, A. Formamidinium Lead Iodide Perovskite Films with Polyvinylpyrrolidone Additive for Active Layer in Perovskite Solar Cells, Enhanced Stability and Electrical Conductivity. *Materials (Basel, Switzerland)* **2021**, *14* (16). DOI: 10.3390/ma14164594. Published Online: Aug. 16, 2021.

(5) Herz, L. M. Charge-Carrier Dynamics in Organic-Inorganic Metal Halide Perovskites. *Annual review of physical chemistry* **2016**, *67*, 65–89. DOI: 10.1146/annurev-physchem-040215-112222.

(6) Guzelturk, B.; Winkler, T.; van de Goor, T. W. J.; Smith, M. D.; Bourelle, S. A.; Feldmann, S.; Trigo, M.; Teitelbaum, S. W.; Steinrück, H.-G.; La Pena, G. A. de; Alonso-Mori, R.; Zhu, D.; Sato, T.; Karunadasa, H. I.; Toney, M. F.; Deschler, F.; Lindenberg, A. M. Visualization of dynamic polaronic strain fields in hybrid lead halide perovskites. *Nature materials* **2021**, *20* (5), 618–623. DOI: 10.1038/s41563-020-00865-5.
